# Quarterly Report of the Obstetric Practice in the Philadelphia Dispensary, for the First, Second, and Third Months, 1841

**Published:** 1841-05-08

**Authors:** J. Warrington


*Quarterly Report of the Obstetric Practice in the Philadelphia Dispensary, for the First, Second, and Third months,* 1841. By J. Warrington, M. D.

Forty-one women have been delivered at full term, of forty-one children,—viz: twenty-four girls, and seventeen boys. The average duration of labour in twenty-seven of these cases, was five hours and thirty minutes; the extremes being one, and thirty hours. The average amount of time occupied in the spontaneous delivery of the placenta, in twentythree cases, was twenty-two and a half minutes, the extremes being ten and sixty minutes.

In twenty-two cases in which the position of the child was carefully noted in the early stage of labour, sixteen were found in the first position, three in the second, two in the fourth, and one in the fifth position of the head.

The case of the fifth position, as well as one of the fourth positions, were converted by the aid of the finger,—the first into a first, and the other into a second position; by which the delivery was more easily accomplished.

One of the children was stillborn, after a very rapid labour, the woman having previously suffered from uterine irritation, and had, subsequently, metritis. The child was slightly shriveled, as though it had been dead some time. In one case, the child presented the anterior fontanelle to the centre of the pelvis—requiring first the use of the vectiss for its correction, and finally the forceps for its delivery. Child did well, and the mother recovered rapidly, with a subsequent attack of pruritis vulva, which was promptly cured by catheterism, and a lotion of subborate of soda, in mucilage.

Two of the women had profuse hæmorrhage after delivery, which, in one case, was promptly arrested by frictions, compressions over the uterus, and ergot. In the other it was controlled after the use of brandy, wine of ergot, active frictions, forcible compressions, and the use of ice in the vagina, and finally, the introduction of a pealed juicy lemon, into the cavity of the uterus. The first case recovered readily, the other, who remained a long time feeble, in consequence of the excessive drainage of blood, and a subsequent metritis, is now recovering very satisfactorily.

There has been a strong disposition to puerperal fever among our patients, during the past three months, and their cases have required an unusual amount of vigilance.

